# Advances in Photonic Gas Sensors Operating in the VIS–NIR Spectrum: Structures, Materials, and Performance

**DOI:** 10.3390/s26051568

**Published:** 2026-03-02

**Authors:** Nourhan Rasheed, Xun Li, Mohamed Bakr

**Affiliations:** 1 Department of Electrical and Computing Engineering, McMaster University, Hamilton, ON L8S 4L8, Canada; rasheedn@mcmaster.ca (N.R.); lixun@mcmaster.ca (X.L.); 2 Department of Electrical Engineering, King Fahd University of Petroleum and Minerals (KFUPM), Dhahran 31261, Saudi Arabia

**Keywords:** photonic gas sensor, silicon photonics, microring resonator, Mach–Zehnder interferometer

## Abstract

The growing need for real-time, accurate monitoring of hazardous gases in environmental, industrial, and healthcare settings has highlighted the limitations of traditional sensing methods. Photonic Integrated Circuits (PICs) have become a revolutionary platform due to their high sensitivity, accurate selectivity, compact size and cost-effectiveness. We present in this work a comprehensive overview of the best-reported PIC-based gas sensors. We discuss the basic concepts behind resonance-based and absorption-based sensing. A detailed overview of the various material platforms, from well-known silicon and silicon nitride to new polymers, chalcogenide glasses, and 2D materials, is presented. A comparison of key device topologies, such as waveguides, microring resonators, Mach–Zehnder interferometers, and metasurfaces, is conducted, with performance benchmarks indicating the limit of detection (LoD). The main limitations of PIC sensors are discussed in this review. We also discuss promising technologies, especially the game-changing potential of artificial intelligence to create fully autonomous devices.

## 1. Introduction

Different applications have different needs when it comes to accurately finding pollutants. Detecting small levels of harmful gases, such as nitrogen dioxide (NO_2_), carbon monoxide (CO), Sulphur dioxide (SO_2_), volatile organic compounds (VOCs), and methane (CH_4_) in parts per billion (ppb), is crucial for maintaining public health and air quality. For industrial safety, on the other hand, the focus is on cost-effectiveness, durability, and the ability to identify harmful or explosive levels, such as finding methane in mines or carbon monoxide in homes. Photonic Integrated Circuits (PICs) are well-positioned to address both of these requirements. They are well-suited for environmental monitoring due to their high sensitivity, and they are also easy to create in large quantities, leading them to be cost-effective for many consumer safety devices [1,2].

Electrochemical and non-dispersive infrared (NDIR) methods have been used for decades to monitor environmental gases [3]. Electrochemical sensors are small, inexpensive, and portable; however, they have several drawbacks, including sensitivity drift, cross-reactivity, and limited lifespan [4]. They work by detecting gases via oxidation reactions at the electrode surface. NDIR devices are more effective at detecting gases like CO_2_. They consume, however, a significant amount of power, and are typically only sensitive to parts per million (ppm) levels, which is insufficient for real-time trace detection in dynamic or indoor environments [5,6].

PIC gas sensors can detect gases with high accuracy and high speed by measuring changes in the light signal absorption or shifts in the light resonance wavelength. To achieve this performance, these sensors exploit nanoscale interactions between light and matter. The complex effective refractive index is measured by the changes in absorption or resonance frequency. Because they use resonance enhancement and nanoscale light confinement, they can detect parts per billion (ppb) levels in real time. Nanoscale confinement maximizes the overlap between the optical field and the gas molecules, while resonance structures propagate light, effectively increasing the optical path length and interaction time within a compact footprint [7,8].

Additionally, typical Complementary Metal-Oxide-Semiconductor (CMOS) compatible technology may be used to produce PIC-based sensors. The fabrication of these sensors can be significantly compact and cost-effective. Production is easily scalable by using these methods. Due to their compact size, multiplexed gas detection and integration with electronics or microfluidics are possible. As a result, the development of portable, low-power environmental sensors becomes feasible. Recent research shows that mesoporous-coated silicon nitride (Si_3_N_4_) Mach–Zehnder interferometers (MZIs) successfully detect VOCs and NO_2_ at ppb levels [9,10]. The PIC sector is still growing quickly due to developments in telecommunications and new sensing applications. Such developments are part of a bigger picture of technological change. Market reports indicate that silicon photonics and PIC-based sensor platforms will increase quickly, making them even more popular in real-world applications [11].

Optical fiber sensors and silica microspheres demonstrate exceptional sensitivity, with long interaction lengths [12] and ultra-high Q-factors [13], but they have discrete, fragile coupling setups, causing them to be harder to integrate into a single system. PICs, on the other hand, use well-established CMOS fabrication methods with distinct advantages in terms of miniaturization, mechanical stability, and the ability to produce large numbers of multi-gas sensor arrays at a low cost per unit. However, this level of integration comes with certain trade-offs. Due to insertion losses between standard fibers and sub-micron waveguides, optical coupling efficiency is still a major problem. Additionally, on-chip resonators are naturally limited by the roughness of the lithographic sidewalls and scattering losses. They usually cannot reach the ultra-high Q-factors (>10^8^) that isolated silica microspheres achieve [14]. Due to the main emphasis on scalable and chip-based sensors, non-integrated platforms are regarded as outside the scope of this review [2].

Given its potential, the body of literature on photonic gas sensing has grown substantially. We looked at more than 150 papers on photonic gas sensing and found 10 review papers that were published between 2020 and 2025. Although these studies examine several aspects of optical sensing, none focus exclusively on integrated gas sensors operating within the visible and near-infrared (VIS–NIR) spectrum.

Butt et al. [15] conducted a thorough review of over 250 articles, and Kazanskiy et al. [16] reviewed over 180 articles to provide general overviews of photonic sensing technologies and their environmental applications. These two reviews covered waveguides and fibres. Tombez et al. [17] focused on methane sensors by reviewing 22 articles. Dinh et al. [3] discussed the NDIR technology by reviewing 40 articles. These are two more specific reviews of gas detection. Butt et al. [18] examined integrated sensors by reviewing 159 articles for detecting toxic gases, while Buckley et al. [19] explored the use of graphene and 2D materials for sensing by reviewing at 406 articles.

Salama et al. [20] investigated the performance of ring resonator-based sensors on silicon platforms by reviewing 62 articles, while Abood et al. [21] provided a full review of how photonic crystal-based gas sensors were developed by investigating over 160 articles.

Jiang et al. [22] recently contributed to the fundamentals of resonance-based gas sensing when they reviewed 140 articles. Kazanskiy et al. [16] discussed new metasurfaces that improve spectroscopy when exploring 48 articles.

Antonacci et al. [23] did a critical review of the Si_3_N_4_ photonics platform for the NIR spectrum by reviewing 38 articles. Yang et al. [24] investigated the performance of the lithium niobate (LN) platform when it is used for creating an optical waveguide by reviewing 153 articles, while Chowdhury et al. [25] reviewed artificial intelligence applications in gas sensing by investigating 216 articles.

Existing reviews have mostly focused on gas sensing in the mid-infrared (MIR) region [15] or specific device architectures like ring resonators [20], leaving a significant gap regarding a comprehensive overview of PICs in the VIS–NIR light spectrum. To address this, we examine integrated gas sensors by applying strict inclusion criteria for chip-scale devices described in recent peer-reviewed literature, while excluding bulk optics, purely MIR devices, and non-optical methods. Although the MIR spectrum offers superior sensitivity via fundamental molecular absorption, the VIS–NIR region is prioritized here due to its distinct advantages in component maturity, high yield, and CMOS-driven cost-effectiveness. We critically analyze how the inherent challenge of weak VIS–NIR gas absorption is overcome through advanced device engineering. We highlight how structural mechanisms, specifically slow-light effects and high-Q resonators, are used to compensate for these low absorption cross-sections by extending the light-matter interaction time [5,26]. Furthermore, we highlight the role of emerging transduction mechanisms, including cavity optomechanics, photoacoustics, and stress-induced sensing, in expanding the capabilities of VIS–NIR platforms. By connecting material platforms with device topology, we explore how emerging platforms such as Si_3_N_4_, Chalcogenides, and LN enable commercially viable, ppb-level detection.

This work is structured as follows: Section 2 outlines the fundamental physical mechanisms of gas sensing. Section 3 details the diverse material platforms employed, evaluating their respective properties and suitability. Section 4 examines the performance of key device topologies implemented across these material systems. Section 5 addresses current fabrication challenges, integration strategies, and future perspectives for environmental and industrial applications. Our findings are concluded in Section 6.

## 2. Fundamentals of Photonic Gas Sensing

**Figure 1:**
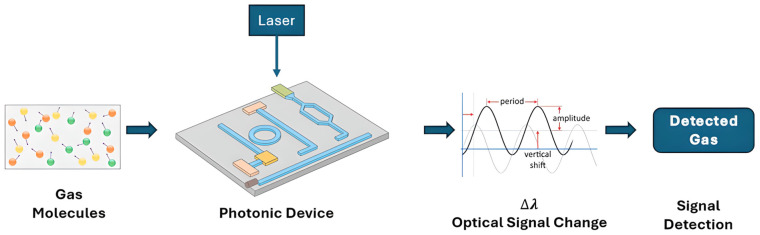
A schematic illustration of the photonic gas sensing process.

Photonic gas sensors utilize the interaction of light with molecules in optical structures to detect the presence of gases and quantify their concentration, as illustrated in Figure 1. This figure is a generic conceptual illustration of how the sensing mechanism works, and it does not depend on any specific material platform or device architecture. It demonstrates the fundamental process in which a light source interacts with gas molecules in a sensing structure, like a resonator, resulting in a detectable change in an output optical signal, such as a change in wavelength, intensity, or phase. These sensors operate on specific physical parameters. Sensing principles are classified based on the primary physical parameter of the guided mode that is disrupted by the gas analyte, thereby offering a deeper physical understanding of the sensing process. We differentiate between mechanisms that change the real part of the refractive index (RI), which causes phase delays or resonant wavelength shifts; those that change the imaginary part of the RI, which causes optical absorption and signal attenuation; and other transduction pathways that use mechanical stress, strain, or thermal changes to cause detectable optical modulation [27,28].

### 2.1. Key Sensing Parameters

To evaluate the effectiveness of photonic gas sensors, it is necessary to measure several key performance metrics. Sensitivity is one of the main metrics. It indicates how much the optical response changes when the medium changes. For sensors that operate based on resonance, this is usually split into gas sensitivity ($s_{gas}$) and refractometric sensitivity ($s_{n}$) [20]:

(1) $$s_{n}=\frac{∆\lambda }{∆n}\;[nm/RIU]$$

(2) $$s_{gas}=\frac{∆\lambda }{∆C}\;[pm/ppm],$$

where Δ*λ* is the shift in resonant wavelength, Δ*n* is the change in the refractive index resulting from the presence of gas, and Δ*C* is the change in gas concentration [15,28,29]. The Figure of Merit (FOM) normalizes the sensitivity against the resonance linewidth, which is the Full Width at Half Maximum (FWHM), to consider a spectral resolution [20]:

(3) $$FOM=\frac{s_{n}}{FWHM}$$

A higher FOM means that the device can deal with small spectral shifts compared to its linewidth.

Selectivity is another important metric of a gas sensor. It measures the sensor’s ability to distinguish the target gas analyte from other gases that might be present in the medium [30]. Optical gas sensors achieve intrinsic selectivity by directly probing the quantized vibrational energy levels of molecules, relying on unique spectral fingerprints to reject background interference. For resonant photonic structures, this selectivity ($S_{A/B}$) is mathematically quantified as the ratio of the resonant wavelength shift ($∆\lambda_{res}$) induced by the target gas (A) relative to an interfering one (B) at equivalent concentrations (C) [31]:

(4) $$S_{A/B}=\frac{∆\lambda_{res}(A)/C_{A}}{∆\lambda_{res}(B)/C_{B}}$$

The LoD is the lowest gas concentration that can be detected [32]. The response time is another metric which measures the time it takes for the sensor to reach 90% of its output in the presence of the gas [33]. Typical values of the response time range from a few seconds to minutes. For a sensor to function properly in the desired workplace, it is essential to optimize these interdependent parameters.

### 2.2. Refractive Index (RI) Sensing

The primary method used to detect wavelengths in the VIS–NIR region is to observe the changes in the real part of the effective refractive index of the guided mode that happen when a gas is absorbed. Two main types of devices can be used to examine this change: Resonators (Spectral Shift) and Interferometers (Phase Shift).

#### 2.2.1. Spectral Shift

Resonator-based sensors exploit the refractometric sensitivity of optical modes to small shifts in the effective refractive index of the surrounding medium. The sensor’s sensitivity measures how much the resonant wavelength changes when the refractive index of the surrounding medium is changed [26]. The basic idea underlying the resonance shift is straightforward. A photonic resonator is an optical device carefully designed to trap and circulate light at a specific wavelength, known as its “resonant wavelength” [34]. When gas molecules in the air around the sensor adhere to its surface, they change how light behaves in that area, specifically the effective refractive index that the light experiences. The resonator is consequently ‘detuned’ by this small perturbation, altering its resonant wavelength. Careful measurement of this shift enables quantification of the ambient gas [18].

Highly effective devices have been created using this principle. For instance, a dual-Fano-resonant silicon metasurface operating at about 1.55 µm was developed in ref. [20]. Significant resonance shifts were reported for CO. The sensor exhibits a good refractometric sensitivity of 1735 nm/RIU for CO, along with good figures of merit, demonstrating how tailored resonance shapes can improve gas selectivity and sensitivity [20,35]. Pushing performance further, recent work on suspended nanomembrane silicon (SNS) microring resonators achieved ultra-high intrinsic quality factors by engineering the waveguide to minimize light scattering, which is crucial for detecting minute spectral shifts [36].

#### 2.2.2. Phase Shift

Interferometric sensors measure the phase shift caused by the gas molecules [37]. The MZI is the most common type of structure for this method. Most MZIs have two paths for the input light: a sensing arm, where the evanescent field is exposed to the gas, and a reference arm, which is protected from outside interference [38].

The principle of operation of interferometric gas sensors is based on the change in the effective refractive index ($n_{eff}$) of the sensing arm that takes place when a gas is absorbed. This change causes a phase shift (Δφ) in relation to the reference arm. When the signals recombine at the output, this change can be measured as a change in intensity [38]. The phase shift is related to the change in the refractive index using the formula [39]:

(5) $$∆\varphi =\frac{2\pi L}{\lambda }∆n_{eff}$$

where λ is the operating wavelength, and *L* is the length of the sensing arm. One of the most beneficial aspects of this method is that sensitivity scales linearly with the sensing length. Researchers use spiral waveguides or folded paths to fit centimeters of optical path length into a small footprint without requiring a larger chip footprint. This approach results in a higher sensitivity [40].

Recent progress has aimed at reducing the size of these structures while keeping their high sensitivity. For instance, Loop-Terminated MZIs (LT-MZIs) use Sagnac loops to reflect light through the sensing arm, which effectively doubles the interaction length for the same physical footprint [38]. Functionalization is also essential in this field of research. For example, covering the sensing arm with polymers or porous materials like mesoporous silica or graphene oxide [23] allows the gas molecules to stay near the waveguide surface. Therefore, the change in the refractive index is larger. Gases like ammonia (NH_3_) and VOCs, as a result, could be detected at ppb levels, as shown in recent graphene-oxide-optimized fibre MZIs [41].

### 2.3. Optical Absorption Sensing

Absorption-based sensors detect the change in the imaginary part of the refractive index. It is known that vibrational overtones control gas absorption cross-sections in the VIS–NIR range, and these values are much lower than those in the MIR fingerprint region. Even though this is a physical limit, looking into absorption sensing in the VIS–NIR is very useful because these sensors are low-cost and utilize non-heated sources and detectors compared to MIR solutions [26]. Furthermore, some dangerous gases, such as nitrogen dioxide (NO_2_), have strong absorption features precisely in the visible spectrum [42].

To compensate for the fact that other gases naturally have weaker overtone absorption, structural engineering is needed to maximize the optical overlap. Since gases in the VIS–NIR range have very low absorption cross-sections, often 10^2^–10^5^ times weaker than MIR fundamental lines, standard waveguide architectures do not always work. In periodic structures such as photonic crystals, slow-light effects decrease the group velocity of light (v_g_). This compresses optical energy and increases the time it takes for light to interact with a unit length. High-Q-factor resonators also trap light, combining a long effective absorption path (L_eff_) into a compact footprint [5,26].

The evanescent field is the tail of the guided optical mode that goes into the medium surrounding it in absorption-based sensors [43]. Gas molecules in the surrounding medium can absorb light at their unique wavelengths. Optimizing this evanescent-field interaction is crucial for the performance of these photonic gas sensors. Recent reviews have emphasized the advantages of complex structures such as slot waveguides and subwavelength-structured (SWG) waveguides, which have been carefully engineered to improve the interaction between light and matter [44]. These designs improve evanescent-field absorption more by strongly confining the light field in the low-index sensing area. Absorption-based sensitivity is thus enhanced by providing more light energy to the area where the gas analyte molecules are, while maintaining acceptable propagation losses [35,45].

The work published in [45] used a slot waveguide to detect CH_4_. The device demonstrated the power and efficacy of strong optical confinement in the slot geometry, attaining a LoD in ppm. Another study employed a chalcogenide hollow-core waveguides [46] has reported a 23 ppm LoD for methane, further supporting this method. A recent theoretical and experimental implementation of an MZI using silicon nitride effectively integrated absorption and sensing [23]. This hybrid method enables simultaneous acquisition of both the real and imaginary parts of the gas analyte’s refractive index, along with a good LoD. These sensing technologies, when combined, can distinguish between complex gas combinations more accurately.

### 2.4. Mechanical and Thermal Sensing

Direct refractive index sensing and optical absorption are among the primary methods by which sensors work in the VIS–NIR region. However, the field has recently grown to include transduction pathways based on mechanical and thermal physics. These other mechanisms offer alternative approaches to deal with the main problem of low gas absorption cross-sections in the VIS–NIR spectrum by turning weak optical interactions into strong mechanical or thermal signals.

Detection methods based on stress and strain rely on the mechanical deformation of the sensing element. Functionalized coatings, particularly polymers, are designed to swell and expand in volume upon absorbing specific gas molecules. The resulting swelling induces substantial mechanical stress and strain on the photonic structure below it (e.g., a Fibre Bragg Grating (FBG) or a microring resonator). This change in shape alters the waveguide’s geometric path length and refractive index through the photoelastic effect, turning a “silent” chemical presence into a measurable spectral shift [47,48].

Thermodynamics can also be used to study the interaction between gas molecules and light. Gas molecules relax without emitting energy after absorbing light, even in the VIS–NIR range. This energy is released as local heat. This thermal signature can be detected either by measuring the change in the refractive index caused by the thermo-optic effect or by using the photoacoustic effect, in which rapid thermal expansion produces pressure waves. High-sensitivity optical resonators subsequently resolve these pressure fluctuations, providing an inherently background-free detection method [49,50].

Optomechanics is the most recent field of sensing. Cavity optomechanics takes advantage of the strong connection between optical fields and mechanical motion in nanostructures. Light is used to read the mechanical vibrations of a small resonator in these systems. When trace gas molecules stick to the surface, they change the resonator’s mass or stiffness. This change in the physical world alters the mechanical resonant frequency, which can then be read with high accuracy using light. This method allows the detection of gases based on their mass and mechanical properties, not just their optical footprint [51].

## 3. Material Platforms for Integrated Photonic Gas Sensing

In photonic gas sensors, functionalization involves adding specific chemical or nanomaterial layers to the sensor’s surface to enable its interaction with a target gas [23]. This method increases sensitivity and selectivity by encouraging the chosen gas analyte to bind. Capture is the process in which specific gas molecules are selectively trapped on the sensor’s surface, which helps reduce interference from other gases. Advancements in material platforms have primarily driven the evolution of photonic gas sensors over the past few years. These materials dictate critical factors such as optical performance, operational spectral range, fabrication scalability, and compatibility with surface functionality. Interferometers, surface plasmon resonance (SPR) devices, microring resonators, photonic crystals, and metasurfaces are all examples of photonic structures that leverage the unique optical and physical properties of these materials to enable more sensitive, smaller, and more selective devices [52]. The primary materials used in these photonic platforms for gas sensing are reviewed here, with an emphasis on their applications in the NIR spectral ranges over the past few years.

### 3.1. Silicon-on-Insulator (SOI)

For a long time, silicon (Si) has been the primary material used in PICs. It has a high refractive index contrast; its refractive index (*n* ≈ 3.45) is much higher than that of the silicon dioxide substrate (*n* ≈ 1.45) used in SOI wafers [26]. The large refractive index difference provides tight optical confinement, which is an essential feature for small footprints. Coupled with its transparency in the NIR spectrum (especially around 1.3 µm–1.6 µm) and compatibility with current CMOS fabrication methods, these properties render it the best platform. These features allow it to be not only cost-effective and easy to scale, but also ideal for developing high-performance miniaturized gas sensors [35].

SOI is even more appealing because it has low optical loss, is thermally stable, and can handle involved waveguide designs. These advantages enable designers to fabricate both passive and active photonic elements, such as modulators, heaters, and splitters, on the same chip. As a result, fully integrated sensing systems become more useful and effective [53].

**Figure 2:**
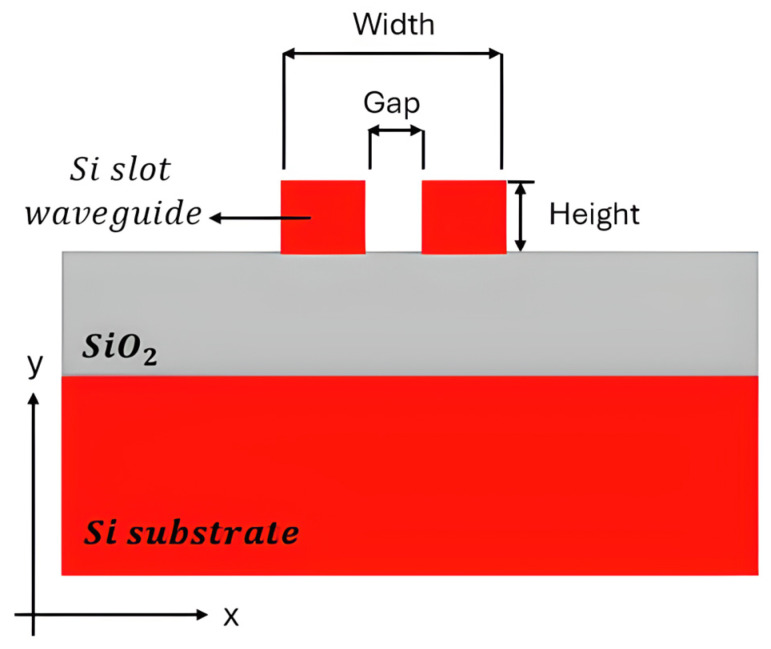
A configuration of a photonic waveguide for biosensing on the SOI platform.

Figure 2 demonstrates the fundamental structure of an SOI slot waveguide platform. For example, a thin silicon layer could be placed directly on an insulator. Alternatively, a buried oxide (BOX) layer is inserted between the guiding layer and the substrate [54]. Structural flexibility is very important for enabling devices to perform well in specific sensing applications. SOI chips can also be easily connected to optical fibers and support 3D integration. Such integration allows for the development of small, plug-and-play lab-on-chip sensing platforms [55,56,57]. In recent decades, SOI has been popular in both research and business. It is one of the most promising areas for developing new photonic gas sensors, which are precise, fast, small, and easy to use in real life [1,2,3].

### 3.2. Silicon Nitride (Si_3_N_4_)

Si_3_N_4_ is a beneficial material in integrated photonics, especially for applications in the VIS–NIR range. It has a wide transparency range, from visible wavelengths to around 6.7 µm. Consequently, it is well-suited for gas sensing over a wide wavelength range, including shortwave MIR (approximately 2–6 µm). That part of the spectrum is particularly valuable because it contains the strong fundamental vibrational absorption lines of many gases. This is often called the molecular fingerprint region, and it provides detection this is much more sensitive than NIR resonances [10,58].

One of the best characteristics of Si_3_N_4_ is that it has very low optical propagation loss across the VIS–NIR spectrum. This high level of transparency is essential when determining trace gas concentrations. It renders Si_3_N_4_ a better and more reliable platform for real-world sensing applications [59].

**Figure 3:**
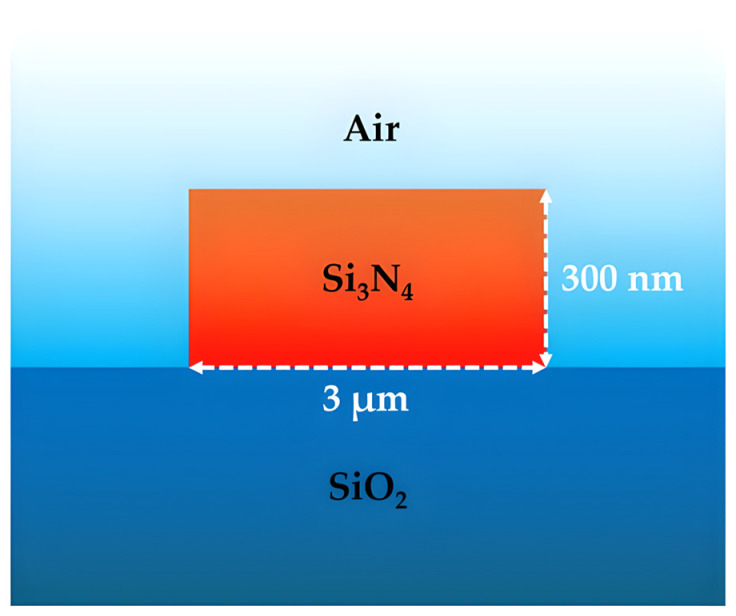
The cross-sectional view of the air-clad Si_3_N_4_ waveguide structure [60].

Recent work on Si_3_N_4_ with a common photonic platform demonstrated its appeal, as illustrated in Figure 3. A recent study showed that a silicon nitride platform worked well at 480 nm, 520 nm, and 633 nm with a single-rib waveguide design. Propagation losses were only 3.6 dB/cm, with tight bending radii (~60 µm) and small multi-mode interferometers [58]. The ability to achieve these metrics demonstrates the advanced nature of Si_3_N_4_ for visible/NIR photonics.

Si_3_N_4_ can support several types of waveguides, including strips, ribs, and slots, as well as various resonator types, including microrings and MZIs. These shapes interact well with thin-film coatings designed to adhere to specific gas molecules. This enables accurate detection of small amounts of gas through evanescent field interactions [61]. Si_3_N_4_ has a somewhat lower refractive index than pure silicon, but the trade-off is worth it for applications that need minimal loss, visible-light operation, or are safe for living beings.

CMOS methods can be used for manufacturing Si_3_N_4_ devices. Unlike silicon, Si_3_N_4_ exhibits a significantly lower temperature sensitivity, which minimizes spectral drift caused by environmental temperature changes. This intrinsic thermal stability reduces the need for complex, power-consuming active temperature control systems, rendering them ideal for robust, low-power, and portable sensing applications [62].

### 3.3. InP and III–V Semiconductors

**Figure 4:**
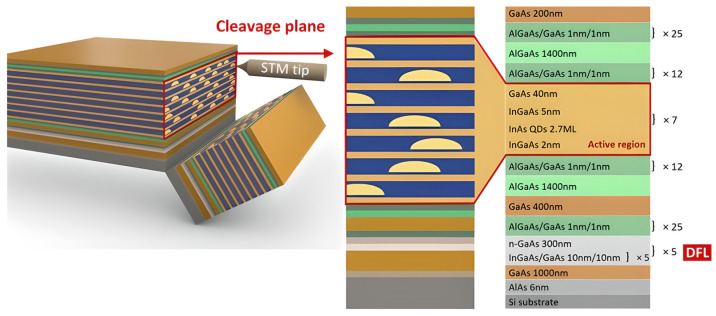
Schematic showing the cleavage plane examined by X-STM/STS and the epitaxial structure of the optimized III–V-on-Si sample [65].

Indium Phosphide (InP) and related III–V compound semiconductors are crucial for the development of photonic gas sensors, as they address the main challenges of silicon-based platforms. Si and Si_3_N_4_ are widely recognized as the premier materials for fabricating passive optical components, offering low propagation losses and high-quality factor resonators [63]. In an indirect bandgap material, the energy maximum of the valence band and the energy minimum of the conduction band are not aligned in momentum space; consequently, for an electron to recombine with a hole and emit a photon, it must simultaneously interact with a phonon (a lattice vibration) to conserve momentum. This inefficient, multi-particle process releases energy primarily as heat rather than light, rendering silicon-based materials unsuitable for active light emission. In contrast, III–V materials possess a direct bandgap where momenta align perfectly, enabling efficient photon emission. This property makes them the industry standard for fabricating the active parts needed to create a complete “spectrometer-on-a-chip,” such as lasers, optical amplifiers, and photodetectors. The main challenge remains how to effectively mount these active III–V components onto passive silicon chips to create a single, compact, and robust sensing device [64]. Recent developments addressed this integration difficulty with two opposing high-performance approaches. The first is monolithic integration, which means growing the III–V material directly onto the silicon wafer, as shown in Figure 4. Crystal defects have long plagued monolithic integration. These defects were addressed using the breakthrough InAs Quantum Dot (QD) lasers [65]. The “defect-tolerant” QDs have enabled the construction of monolithic silicon lasers that perform just as well as those on their primary substrate. Such a breakthrough is a significant step for portable applications, as it demonstrates continuous-wave (CW) operation up to 65 °C on silicon without active cooling. This performance is fundamentally enabled by this material’s active region design. This design spatially separates electrons and holes to significantly suppress the recombination losses to the primary barrier of the uncooled operation in MIR semiconductor lasers. Utilizing the monolithic method, these Interband Cascade Lasers (ICLs) have been successfully implemented on silicon substrates to operate at an emission wavelength of 3.4 µm [63].

The second technique, heterogeneous integration, involves connecting prefabricated III–V devices to silicon. A significant advancement is a 3D self-aligned flip-chip method that employs electrical surface tension to place two Quantum Cascade Lasers (QCLs) with sub-micron accuracy. Portable MIR light generation at 7.2 µm is thereby enabled, at a size 100 times smaller [66].

The integration method extends beyond light sources to enable the entire sensing system to function. The study [67] shows that InGaAs photodetectors may be grown directly on large, 300 mm CMOS-compatible silicon wafers. This approach is essential for NIR sensing (900–1700 nm). It enables the cost-effective, large-scale production of sensors for AI-enhanced analysis and industrial safety [68]. The same “III–V-on-silicon” idea is being used on other materials as well, such as Gallium Nitride (GaN), which enables the development of completely integrated photonic circuits that can sense visible light [69].

### 3.4. Hybrid Platforms: Graphene and 2D Materials

2D materials such as graphene cannot function as the main light-guiding medium in VIS–NIR sensing. Silicon and Si_3_N_4_ are two examples of bulk waveguide materials. Instead, graphene can be added as a functional overlay to dielectric waveguides to create hybrid modes [70]. This greatly improves the interaction between the evanescent field and the surrounding medium because they have a high surface-to-volume ratio [71].

Before chip-scale implementation, the fundamental sensing capabilities of graphene were rigorously established on optical fiber tapers [72] and silica microspheres [73]. These platforms demonstrated that graphene’s high surface-to-volume ratio significantly enhances the evanescent field interaction. While these non-integrated devices fall outside the scope of this review, their physical findings provide the essential justification for transferring 2D materials onto scalable PIC architectures to replicate this high performance in a compact footprint.

Some other 2D materials, such as molybdenum disulphide (MoS_2_) and tungsten disulphide (WS_2_), are receiving significant attention as strong tools for modulating light through surface interactions. Because they are so thin, they have a large surface area relative to their volume, so light and matter interact strongly. Because of this property, molecules can stick to surfaces, which can cause measurable optical or electrical responses, even when the gas concentrations are very low [19,74]. MoS_2_ and WS_2_ are two of the most attractive 2D materials. They are both transition metal dichalcogenides (TMDs). In monolayer conditions, they have direct band gaps that interact with light, altering how light is absorbed. Some studies show that WS_2_ has a higher adsorption capability for certain molecules than MoS_2_. Experimental characterization has also shown that WS_2_ exhibits rapid adsorption kinetics, rendering it a good choice for fast-responding active layers [75].

Graphene exhibits exceptional mechanical strength and high carrier mobility; however, it lacks an intrinsic bandgap. This ‘zero-gap’ characteristic renders its electronic properties highly sensitive to surface perturbations. Upon exposure to analytes, molecules adsorb onto the graphene surface and induce charge transfer. These variations in carrier density shift the Fermi level, thereby modulating both the electrical conductivity and the optical absorption. A mixed structure or hybrid composed of graphene and transition TMDs takes advantage of graphene’s ability to conduct electricity and the ability of WS_2_ or MoS_2_ to absorb light [76,77].

However, these bare materials are susceptible to environmental degradation. This is because they have a large surface area, which makes them more likely to oxidize and be damaged by moisture. Recent studies have shown that passivation methods, such as placing stable graphene overlayers over sensitive 2D layers such as germanium arsenic (GeAs), may help. This method stops environmental damage and protects important properties from fast degradation [78].

Chemisorption is the main interaction mechanism for Graphene [79]. Density Functional Theory (DFT) modelling has shown that a large charge transfer between the gas molecule and the graphene sheet causes this process. By adding transition metals like nickel or cobalt to graphene, this interaction can be made selective for certain molecules. The DFT-calculated charge transfer alters the carrier density of graphene, which, in turn, alters its complex refractive index. Integrating functionalized graphene into a PIC, such as an MZI, converts this optical change into a measurable signal. Consequently, a robust optical platform that works on a chip scale is achieved [80].

### 3.5. Plasmonics: Surface Plasmon Resonance Platforms

Plasmonic sensors have shown outstanding potential in the last few years for rapid and precise molecular detection [81]. The phenomenon of localized surface plasmon resonance (LSPR) measures the extent to which molecular adsorption alters the resonance wavelength, considering the plasmon decay length and surface susceptibility [82]. These platforms often use noble metals like gold (Au), silver (Ag), and aluminum (Al) because they support SPR and LSPR. These resonances give rise to strong, localized electromagnetic fields at the interfaces between metals and dielectrics. As a result, changes in the refractive index can be detected upon gas adsorption. This framework enables comparisons of different materials and shapes, such as dense arrays of gold nanoparticles that exhibit strong field enhancements [82].

Recent research investigated multilayer and composite designs to improve performance. Structures such as patterned plasmonic arrays utilize Au/Ag nanoparticles [83]. They exhibit easy fabrication, multi-gas detection capabilities, and potential applications in environmental and biomedical fields. Nanoporous gold films greatly improve optical field confinement because they have a large surface-to-volume ratio, which further enhances the localized plasmonic field [84,85]. A surface can be selective for certain target molecules by adding functional groups to it or by stacking molecular layers. Recent reviews have shown that coating plasmonic nanostructures with functional materials such as Metal–Organic Frameworks (MOFs) or amine-based receptors enables distinguishing between chemicals more accurately by filtering out interferents and binding to the target analyte more strongly [86].

Tuning the material further enhances its optical properties. Combining silver-based platforms with 2D materials like MoS_2_ results in stronger electromagnetic confinement and higher refractive index contrast [87]. Aluminum-based SPR platforms are less chemically stable, but they support sharper resonances and higher spectral resolution [82]. Current research on hybrid alloys, such as Au–Ag alloys combined with WS_2_, demonstrates strong light-matter interactions and sharp spectral features, confirming the capability of these nanostructures for advanced optical applications [88].

### 3.6. Lithium Niobate (LN)

Lithium niobate (LiNbO_3_ or LN) has become one of the most powerful materials in integrated photonics because it has a strong Pockels electro-optic effect [89]. This linear electro-optic phenomenon allows the refractive index of the material to be modified linearly in proportion to an applied electric field, enabling ultra-fast modulation and precise spectral tuning of the optical field [90]. It has a wide range of transparency from ultraviolet to the MIR and demonstrates exceptional thermal and mechanical stability. These features allow fast and easy control of on-chip photonic components, thus explaining the reason why LN is an excellent material for advanced tunable photonic devices [91].

**Figure 5:**
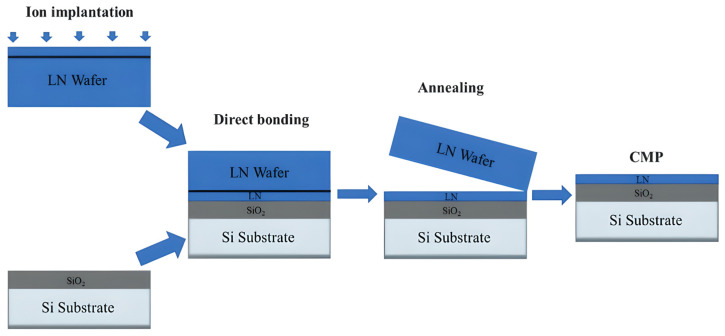
An illustration of the fabrication process of lithium niobate on insulator [91].

Recent studies have utilized electro-optic (EO) dual-comb spectroscopy for high-resolution on-chip spectral analysis. In this mechanism, two frequency combs with slightly different repetition rates are generated using LN modulators. These combs interfere to down-convert the optical absorption spectrum of the gas into the radio-frequency (RF) domain, allowing for rapid, high-resolution measurement of gas absorption lines without tunable lasers [91]. Figure 5 shows the fabrication process of a typical LN device using wafer bonding.

The development of high-quality LN thin films generated via economical sputtering, as opposed to ion implantation or smart-cut methods, has increased fabrication challenges while facilitating scalable and low-loss LN photonic devices [24]. The Lithium Niobate on Insulator (LNOI) platform has recently become a strong solution for heterogeneous integration. It renders it possible to develop CMOS-compatible photonic circuits that combine the electro-optic properties of LN with the ability to scale silicon processing [92].

Combining LN with Si_3_N_4_ in a hybrid way enables the fabrication of wafer-bonded devices. The rationale is that Si_3_N_4_ provides low-loss guiding, while LN offers fast electro-optic tuning. LN microring resonators feature real-time voltage-controlled resonance tuning for dynamic spectral matching [93]. Their optical properties additionally render them useful for rapidly interpreting signals and developing small integrated optical circuits [91].

### 3.7. Polymers

Polymers such as polymethylmethacrylate (PMMA) and epoxy-resin-type materials (SU-8) are becoming increasingly appealing for photonic platforms due to their low cost and flexibility. They are also compatible with scalable manufacturing technologies, including spin-coating, hot embossing, and nanoimprinting. PMMA is transparent from visible to NIR wavelengths and has a refractive index (*n* ≈ 1.48). SU-8, on the other hand, has a higher refractive index (*n* ≈ 1.57) and can support high-aspect-ratio structuring, which is excellent for patterned optical components and slot waveguides [94,95].

Polymers such as PMMA have become desirable platforms because they are flexible and can be used to fabricate large areas at low temperatures. Because they are easy to work with, compact waveguide architectures with integrated functional coatings can be developed. Metal–organic frameworks (MOFs) or polymers, such as polyhexamethylene biguanide (PHMB), significantly enhance molecular adsorption and selectivity. Optical platforms incorporating MOFs enable light to interact with molecules inside a thin MOF layer. For example, PMMA ridge waveguides covered with zeolite imidazole framework-8 (ZIF-8) MOFs maintain low optical losses while facilitating rapid adsorption of energy. MOF-coated planar polymer structures also exhibit stable, reversible molecular interactions, showing that polymers can be both sensitive and reliable in real-world functional applications [96].

Polymer-based waveguides have achieved enhanced optical properties through structural innovation and hybrid integration, surpassing those of traditional ridge designs. Advanced setups, such as bimodal SU-8 waveguides with plasmonic aluminum stripes and slot-enhanced geometries, offer strong light confinement that rivals silicon-level performance but at a much lower cost [95,97]. The implementation of complex geometries, such as spiral SU-8 waveguides or Bragg gratings filled with functional materials, further maximizes the interaction volume [40]. These improvements demonstrate that polymer photonics is a flexible, scalable approach for developing high-performance, compact photonic devices with adjustable surface chemistry and enhanced optical confinement.

### 3.8. Chalcogenide Glasses (As_2_S_3_)

Chalcogenide glasses (ChGs), such as arsenic trisulfide (As_2_S_3_), are excellent materials for integrated MIR photonics because they are highly transparent from the visible range to over 10 µm, including important gas absorption bands. Their high refractive index (*n* ≈ 2.4) and large third-order nonlinearity (*n*_2_ ≈ 2–5 × 10^−18^ m^2^/W) enable them to confine light strongly and create efficient nonlinear optical effects in small areas [98]. These material characteristics make ChGs ideal for on-chip spectroscopic platforms operating in the MIR.

The strong nonlinear properties of on-chip As_2_S_3_ waveguides can generate a supercontinuum extending deep into the MIR, making them suitable as integrated broadband light sources for spectral analysis [99]. Furthermore, femtosecond laser fabrication has enabled the fabrication of high-quality (Q~10^6^) As_2_S_3_ microresonators that can be tuned thermally and optically. Chalcogenide photonic crystal fibers and suspended-core fibers have also demonstrated broad supercontinua spanning 1.5 to 8 µm, highlighting the material’s strong mode confinement and wide MIR transparency [100,101].

Hybrid integration of ChG photonics with standard silicon platforms enables them to work better. Using low-temperature methods like thermal evaporation or sputtering, amorphous ChG films can be directly deposited on pre-patterned silicon circuits. Because they do not require lattice matching, these glasses can be integrated into the back-end of CMOS processes without damaging existing electronic components [101]. The high nonlinearity of ChGs additionally supports Brillouin lasing and two-octave supercontinuum generation [102,103]. The broad transparency window, high refractive index, and flexible low-temperature fabrication of chalcogenide materials make them CMOS compatible and a highly adaptable platform for integrated broadband spectroscopy [104].

### 3.9. Summary of Material Platforms

Table 1 provides the main optical properties, fabrication limitations, and advantages of the material platforms addressed in this section. Choosing an appropriate material means finding a balance between the desired operational wavelength, the needed optical confinement, and the ability to produce it in large amounts.

## 4. Photonic Gas Sensor Structures

This section provides an overview of how photonic gas sensors work at their fundamental level. The designs are based on geometric structures that improve light-gas interactions, resonant and filtering structures that detect small changes in light, and interference-based systems that use phase-sensitive measurements. The goal is to highlight how each approach manipulates light to achieve high sensitivity and selectivity for gas detection [55]. Most of these architectures depend on guided light propagation, so the optical waveguide is the most crucial component that holds and directs light, allowing the optical field to interact with the gas analyte surrounding it [105].

**Figure 6:**
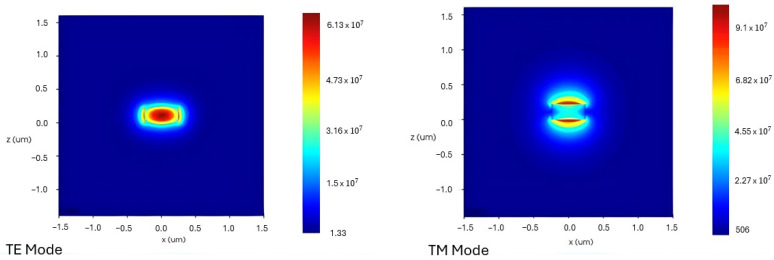
The 2D electric field representation of TE (**left**) and TM (**right**) modes profiles of a SOI rib waveguide on SiO_2_ substrate [106].

A waveguide, like an optical fiber, is a structure formed to direct electromagnetic energy within a specific volume, as shown in Figure 6 [106]. The cross-sectional geometry of the structure plays a crucial role in how light travels through it by determining the supported modes. Each mode represents a transverse field distribution with a specific polarization and amplitude along the direction of propagation [107]. Transverse Electric (TE) and Transverse Magnetic (TM) modes are often used. The difference between them is whether the electric or magnetic field stays perpendicular to the direction of propagation [107,108]. It is important to control these polarization states for sensing. Recent advances, like compact TM-mode filters in rectangular-air-hole 1D photonic crystal waveguides, have shown that this is possible by achieving high polarization extinction ratios. Waveguides can be built in many ways. For example, one-dimensional (1D) photonic crystal waveguides use periodicity to control the flow of light [109]. Two-dimensional materials like graphene have a layered structure and a large surface-to-volume ratio, which renders them suitable for gas detection [110]. In the end, it is essential to carefully tune the waveguide design, including its cross-section, dimensions, material platform, and modal polarization. Maximizing the overlap between the optical mode and the gas analyte molecules through this tuning improves the sensitivity and performance of photonic gas sensors [107,109].

Table 2 presents a comprehensive overview of the three main photonic architectures used in VIS–NIR gas sensing by showing their physical mechanisms and structural trade-offs.

### 4.1. Waveguide-Based Photonic Gas Sensors

Photonic gas sensors rely on waveguides to direct light while extending an evanescent field into the surrounding medium, enabling gas molecules to either absorb specific wavelengths or alter the effective refractive index. Various waveguide geometries are discussed, such as rib, slot, and photonic-crystal designs, to enhance this light–matter interaction by increasing field overlap with the gas analyte and improving sensitivity [110]. The following section highlights key advances in photonic waveguide gas sensors across different material platforms, showing how diverse structures are continually optimized for higher performance.

#### 4.1.1. Silicon-Based Platforms (SOI)

**Figure 7:**
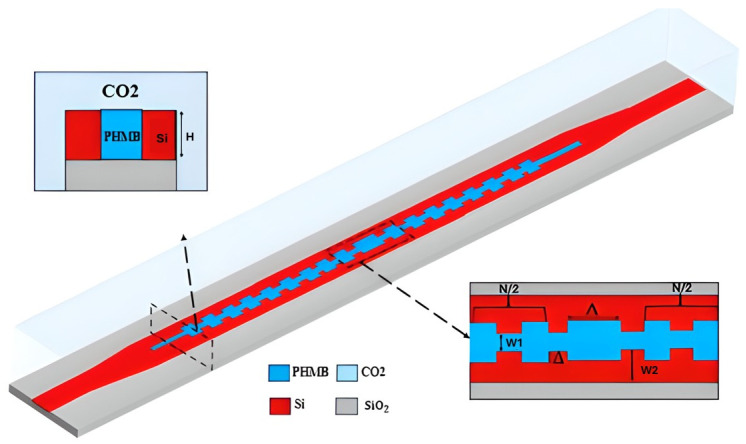
The structural schematic of the phase-shifted Bragg grating slot waveguide [111].

A compact CO_2_ sensor that uses an SOI slotted polymer–phase-shift Bragg grating (SP-PSBG) waveguide integrates PHMB directly into a 220 nm-thick slot between silicon rails that are 100 nm separated, as shown in Figure 7. The PHMB-filled slot allows light to interact with the gas analyte, and the 12 nm sidewall corrugations and central defects provide a sharp resonance for accurate detection. The device has a gas sensitivity of 14.4 pm/ppm, a narrow 1.6 nm full width at FWHM, and a FOM of 9 × 10^−3^ ppm^−1^, which is better than regular polymer-coated sensors. The 38.2 µm footprint shows how well hybrid silicon–polymer waveguides work for small, high-resolution gas sensing [111].

In addition to these hybrid-polymer methods, designing the shape of the basic silicon waveguide to improve gas sensing remains a significant area of research. A theoretical study examined the fundamental properties of silicon-based ridge waveguides for trace gas detection at a wavelength of 1392 nm [112]. The design was optimized to maximize evanescent-field absorption. By carefully designing the waveguide’s modal dimensions, an evanescent field ratio (EFR) exceeding 30% was achieved, ensuring significant interaction between light and matter. The outcome is high theoretical sensitivity and a good LoD of 26 ppm.

Experimental work has confirmed this methodology, showcasing on-chip absorption spectroscopy utilizing an extended SOI waveguide. One CH_4_ sensor used a 10 cm-long silicon waveguide in a small “paperclip” shape to monitor the NIR absorption line at 1650 nm. The system used the evanescent optical field of the high-index-contrast waveguide to achieve a LoD of less than 100 ppm in the presence of Gaussian noise. This LoD is higher than that of MIR alternatives. However, it demonstrates that chip-scale silicon photonics can be utilized for small, cost-effective gas monitoring [17].

#### 4.1.2. Silicon Nitride (Si_3_N_4_)

**Figure 8:**
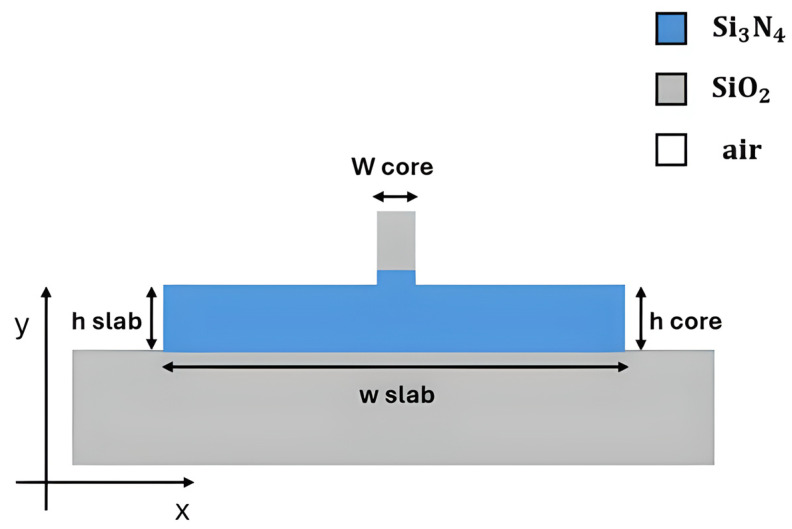
An illustration of a Si_3_N_4_ rib waveguide cross-section.

The silicon nitride-on-silicon dioxide (SiNOI) platform is suitable for an MZI-based sensor across a wide spectral range. A small sensor was developed for use with visible light by using a loop-mirror-terminated (LMT) MZI with a 150 µm slot waveguide sensing arm. The device worked at 650 nm and had a refractometric sensitivity of about 1320 nm/RIU and a FOM of 641 RIU^−1^ for this type of setup [113]. The platform is also powerful in the NIR range, where an ultra-sensitive sensor achieved ppb detection using an MZI with long (~5 mm) interference arms and a mesoporous silica functionalization layer. The resultant combination produced an unprecedented LoD of 1.6 ppb for ethanol and 65 ppb for acetone, taking advantage of the Si_3_N_4_’s larger evanescent tail, which allowed for stronger light-matter interaction [23]. Resonant waveguide gratings are another type of interferometer. A Si_3_N_4_ rib waveguide, as illustrated in Figure 8, with a sinusoidally modulated width, was used to create a long-period grating (LPG), which showed a high-sensitivity sensor. The structure operates at about 1550 nm and couples light to modes that strongly affect the medium around them. It has a high refractometric sensitivity of 11,500 nm/RIU, which shows that it could be used for high-performance gas sensing when it is functionalized [114].

#### 4.1.3. Chalcogenide Glass

**Figure 9:**
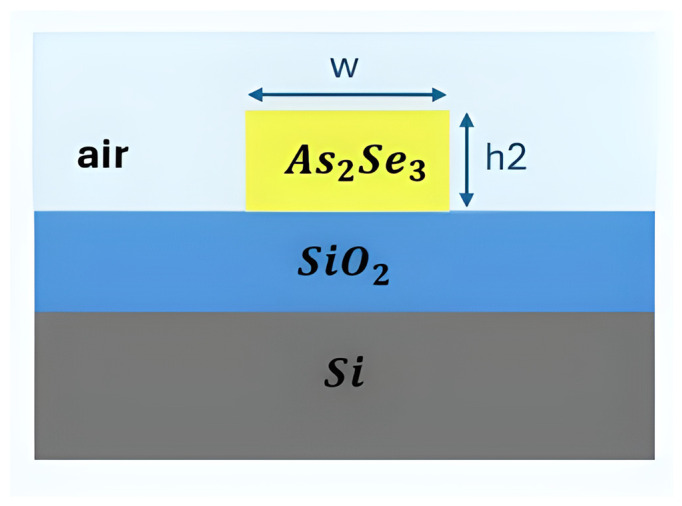
A 2D illustration of the slotted ChG/SiO_2_ waveguide sensor.

Chalcogenide glass (ChG) is a key material for gas sensing, and improvements in its fabrication are needed to enhance its performance. Figure 9 shows a gas sensor fabricated using the ChG material [103]. Adding argon (Ar) to fluorine (CHF_3_) in an optimized reactive ion-inductively coupled plasma (RIE-ICP) etching process for solid-core GeSbSe waveguides cuts propagation losses in the NIR by a significant amount, bringing them down to 2.6 dB/cm at 1.55 µm [115].

ChG has also been used in a novel manner as the anti-resonant layer in hollow-core structures [46]. These structures trap light in a gas-filled core, resulting in a large external confinement factor (ECF) of about 71%. The polarization-insensitive design [46] was able to detect acetylene at 1.532 µm and CH4 at 1.654 µm, with a best LoD of 23.07 ppm.

#### 4.1.4. Lithium Niobate (LN)

**Figure 10:**
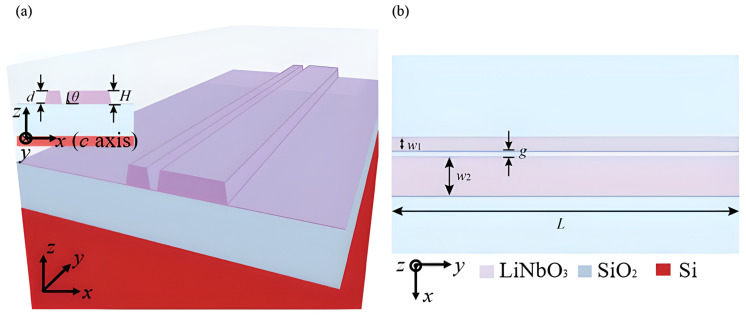
The Si–LN hybrid waveguide: (**a**) the working mechanism and (**b**) the corresponding cross-sectional schematic of the waveguide structure [118].

Lithium niobate on insulator (LNOI) is becoming a popular platform for gas sensing because it has strong electro-optic and nonlinear interactions, very low losses (0.34 dB/m at 1550 nm), and high Q-factors (up to 10^8^) [91]. Photothermal (PT) spectroscopy is an advanced sensing technique that uses an LNOI rib waveguide to heat CO_2_ with an MIR pump (2004 nm) and then detect the change in the effective refractive index with a NIR probe (1550 nm). Figure 10 shows an LNOI rib waveguide. The approach has an 870 ppm LoD [116]. Another technique is to use second-harmonic generation (SHG) in a periodically poled (PPLN) waveguide to turn a 1550 nm NIR pump into 775 nm visible light. Gas adsorption alters the effective refractive index, leading to a shift in the phase-matching of output intensity at a fixed wavelength, giving good sensitivity [117]. LN is suitable for dynamic sensors because it has a unique combination of electro-optic, piezoelectric, and acousto-optic properties. However, for it to be widely used, high-quality sputtered thin films that can be scaled up are needed to replace wafer-bonded layers [91].

#### 4.1.5. Polymer and Organic Material

**Figure 11:**
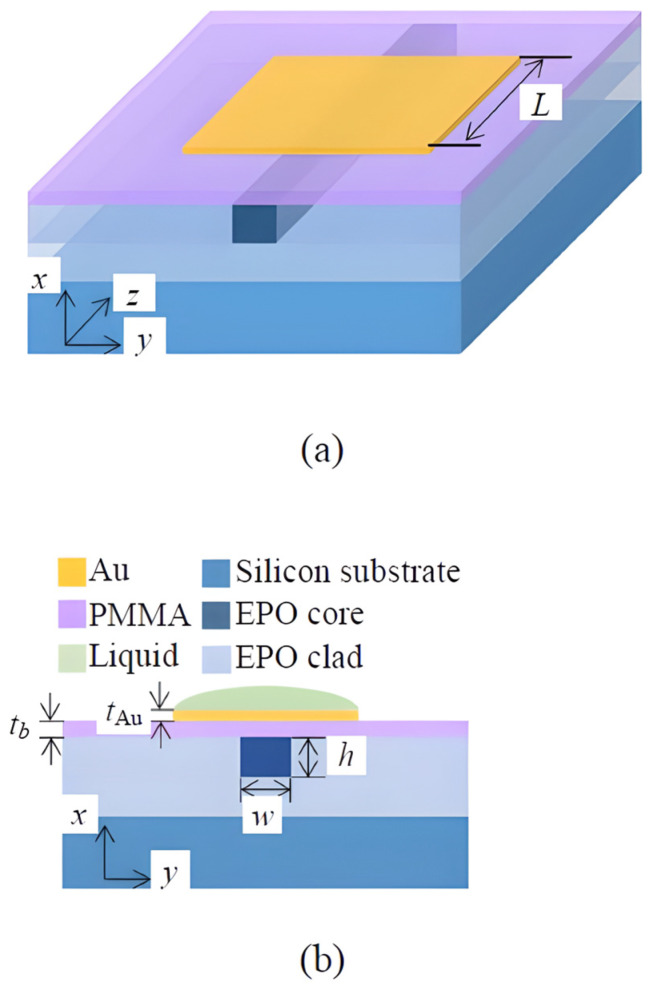
A schematic of a sensor utilizing polymer waveguide platforms: (**a**) 3D representation of the waveguide geometry and (**b**) the corresponding cross-section of the waveguide [119].

PMMA, SU-8, and BCB are becoming increasingly popular for photonic gas sensing because they are cheap, flexible, and work well with manufacturing technologies that can be scaled up, as shown in Figure 11 [94,95]. Their ease of processing allows for the integration of functional coatings to improve selectivity, while structural innovations have made polymer waveguides highly sensitive.

By leveraging structural innovation, polymer waveguides have also become highly sensitive. The first on-chip detection of NIR acetylene in the telecom band [40] was enabled by advanced designs, such as a spiral SU-8 waveguide. Additionally, bimodal SU-8 waveguides have reached refractometric sensitivities of 6300 nm/RIU by using high-confinement geometries. Slot-enhanced geometries have reached an impressive 2.39 × 10^5^ nm/RIU [95,97]. These improvements demonstrate that polymer photonics is a flexible, scalable approach to developing low-cost, high-performance gas sensors.

#### 4.1.6. Hybrid Platforms: Graphene and 2D Materials Integration

**Figure 12:**
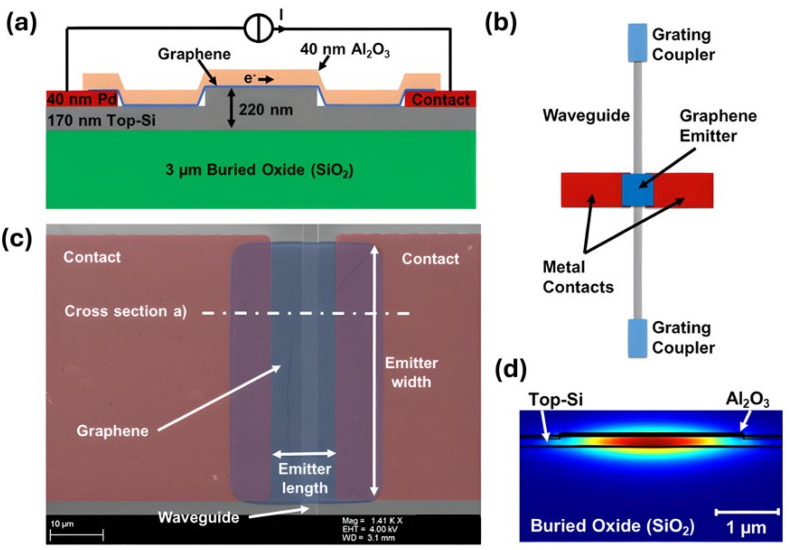
Device layout and waveguide simulation: (**a**) cross-sectional schematic of the encapsulated graphene waveguide-integrated thermal emitter, showing the electrical connections used for Joule heating-induced thermal emission, (**b**) top-view schematic of the PIC components, (**c**) false-colored scanning electron micrograph of the device before encapsulation, and (**d**) simulated electric field distribution of the fundamental TE mode within the waveguide, highlighting the constituent materials [120].

Combining graphene and 2D materials with photonic waveguides enables the creation of a wide range of active and passive gas sensing mechanisms. The creation of an on-chip thermal emitter [120] is an important step forward for active components. It uses graphene directly on a silicon waveguide to act as a light source for on-chip spectroscopy, as illustrated in Figure 12. The structure is a step forward towards integrated CO_2_ detection. Two-dimensional materials are also excellent passive sensing layers. For example, a numerical study for a visible and NIR sensor suggested an SPR structure based on a graphene-gold (Au)-silicon carbide (SiC) waveguide. The graphene overlayer in this design enhances molecule adsorption and boosts the evanescent field, thereby greatly increasing the sensor’s predicted sensitivity [121]. The passive method has also been tested in the lab, where a silicon waveguide covered with a few layers of MoS_2_ was used to find NO_2_. In this device, the absorption of guided light is changed by the adsorption of NO_2_ molecules onto the MoS_2_ surface. Minimal changes can be detected in the waveguide’s output power [122]. These examples demonstrate the flexibility of 2D materials in waveguide-based systems, where they can serve as active emitters or passive layers that enhance absorption or plasmonic-photonic interactions.

### 4.2. Resonator/Filter-Based Photonic Gas Sensors

Microring resonators and metasurfaces are examples of photonic resonators that confine light. This creates an effective refractive index shift when gas molecules are present. The interaction causes the device’s spectral response to change, as measured, for example, by a change in its resonant wavelength. The high-quality (Q) factor of these structures is an important and essential feature for resonant structures. Narrow resonance linewidths lead to high sensitivity and the detection of very low gas levels [22]. This section discusses major improvements in resonator-based sensors, organized by the primary materials used and the various ways they are used.

#### 4.2.1. Silicon on Insulator

**Figure 13:**
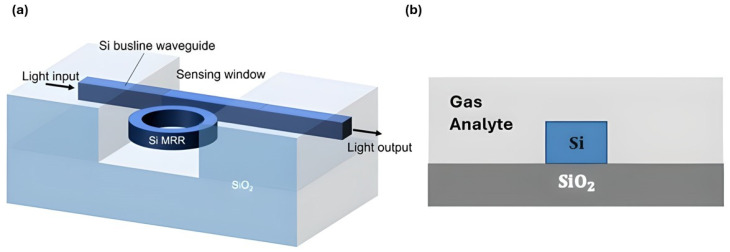
(**a**) A 3D perspective view of the design of the MRR, and (**b**) a cross-sectional view of the structure [129].

SOI microring resonators (MRRs), as illustrated in Figure 13, are a well-established and effective technology for gas sensing in the NIR range. An MRR works by sending light from a straight bus waveguide into a circular ring. This traps certain wavelengths, which creates sharp optical resonances. When a target gas interacts with the optical evanescent field around the ring, it changes the effective refractive index, which causes a noticeable and measurable change in the resonant wavelength [123]. MRRs are small, have high Q-factors, and are compatible with CMOS technologies. One common way to guarantee that a sensor is selective is to functionalize its surface. Researchers are developing more complex structures to enhance the interaction between light and matter, ultimately rendering them more sensitive. For example, a new design combines a slot and a Bragg grating into a “Grating Slot Micro-Ring Resonator” (GSMRR) on the SOI platform. This approach is intended for high-efficiency gas sensing in the 1550 nm range [124]. For NIR gas detection, advanced setups with multiple resonators are also under development, such as double-ring resonators (CROWs) [125]. Another example of microring resonators developed using SOI has shown excellent sensitivity for gases like NO_2_, with a LoD below 20 ppb, which is an essential level for many environmental monitoring applications [126]. It is possible to design cascaded systems that exploit the Vernier effect [127], which significantly increases overall sensitivity beyond that of a single ring. A study developed a parallel-ring MRR structure to solve the significant issue of cross-sensitivity. The design utilizes two rings of different widths to simultaneously measure temperature and refractive index, allowing the sensor to distinguish the gas signal from changes in environmental temperature [128].

#### 4.2.2. Silicon Nitride (Si_3_N_4_)

**Figure 14:**
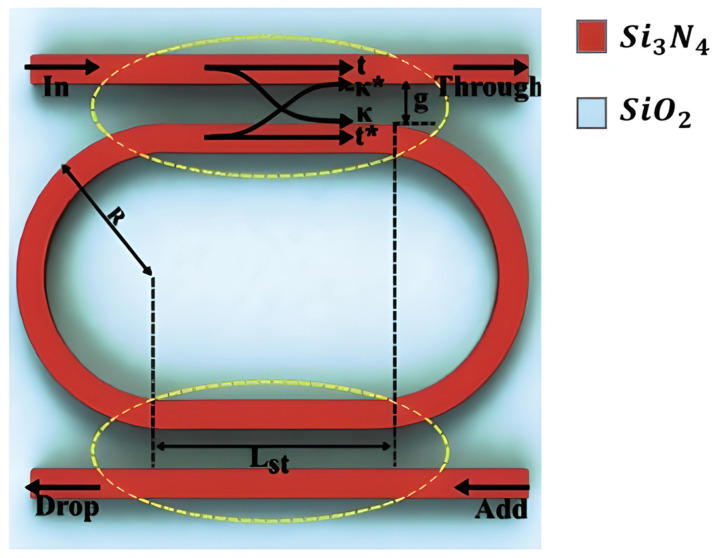
Schematic of a Si_3_N_4_ add-drop racetrack MRR on silica. *t*, *κ* and t*, κ* denote the self- and cross-power coupling coefficients of the propagating fields, respectively [131].

**Figure 15:**
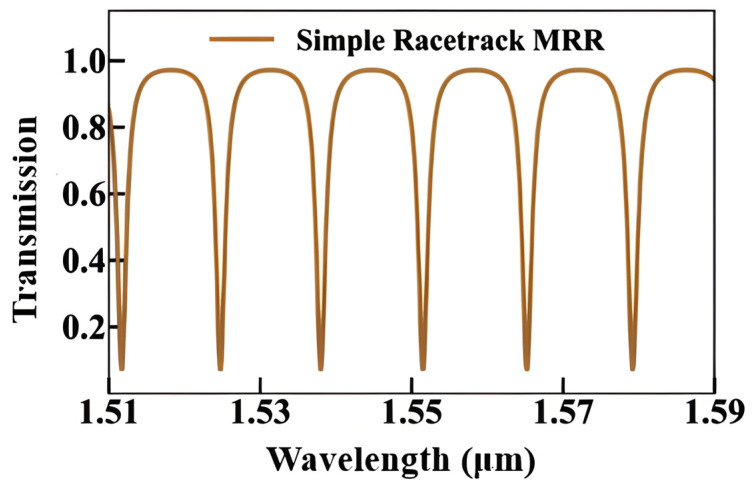
A transmission spectrum of the Si_3_N_4_ racetrack resonator schematic [131].

Si_3_N_4_ is a highly efficient platform for resonator-based sensors because it has low propagation losses, which enable high-Q factors, and broad transparency from the VIS to the NIR spectrum [130]. Initial research on Si_3_N_4_ race-track resonator-based sensors showed that sensor sensitivity varies with wavelength, as illustrated in Figure 14. A study integrating numerical analysis and experimental validation within the 1520–1600 nm range revealed that the refractometric sensitivity rises from 116.3 nm/RIU to 143.3 nm/RIU at extended wavelengths by using the Si_3_N_4_ racetrack resonator structure. It also proved that this design has a good transmission response, as shown in Figure 15 [130]. Other studies [130] have numerically and experimentally demonstrated the use of Si_3_N_4_ ring resonators for refractive index sensing, attaining comparable sensitivities of approximately 112.5 nm/RIU. One of the main issues with these basic refractometric sensors is their lack of selectivity. They cannot differentiate between gases with similar refractive indices [130]. Functionalization is one of the primary methods to solve this problem. A study [23] showed that coating a Si_3_N_4_ photonic resonator with a mesoporous silica layer improved selectivity. This developed a gas sensor with a LoD that could detect ethanol and acetone at levels as low as 1 ppb.

#### 4.2.3. Polymer and Organic Materials

**Figure 16:**
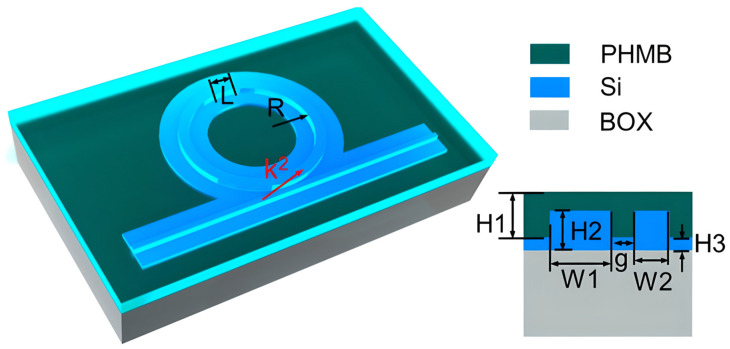
Cross-sectional schematic of the silicon racetrack MRR covered by a polymer layer, illustrating the 220 nm top silicon thickness (H2) and 150 nm etched rib profile [134].

**Figure 17:**
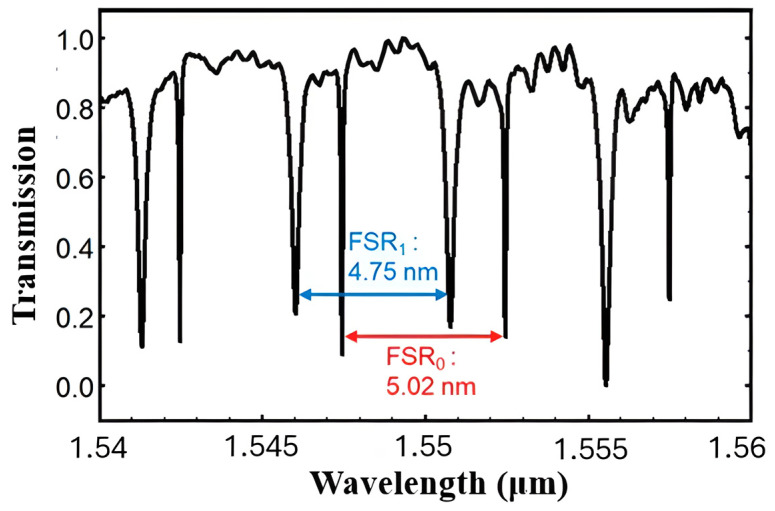
The normalized transmission spectrum of the microring resonator with a PHMB-coated polymer [134].

All polymer devices are a low-cost and flexible solution for resonator-based sensing. They use properties such as biocompatibility and compatibility with scalable manufacturing methods, including 3D microprinting and inkjet printing [132,133]. For example, a silicon photonic MRR was coated with a PHMB polymer layer to create a refractometric sensor for CO_2_, as illustrated in Figure 16 [134]. This hybrid device had a LoD of 20 ppm and excellent transmission response, as shown in Figure 17. The all-polymer whispering-gallery-mode (WGM) microdisk resonator is an excellent example of using SU-8 polymer to detect VOCs [132]. These polymers often have a dual-factor sensing mechanism. One factor is the change in the material’s refractive index when it detects gas, and the other is the material’s physical swelling, which changes the resonator’s radius [132]. This dual mechanism is not limited to SU-8. A study [135] of a self-assembled Polydimethylsiloxane (PDMS) microbottle resonator similarly utilizes both the refractive index and volume change to detect ethanol gas, achieving a high sensitivity of 36.24 pm/ppm [136]. One interesting feature of these platforms is that they use geometric design to improve performance. For example, by creating a larger microdisk undercut, the structure can swell more freely, resulting in a more resonant shift [132]. Another study that used drop-on-demand inkjet printing to create WGM resonators from several polymers (including SU-8 and PHMB) for detecting gases such as CO_2_ demonstrated the process’s flexibility [133].

#### 4.2.4. Dielectric Metasurfaces

**Figure 18:**
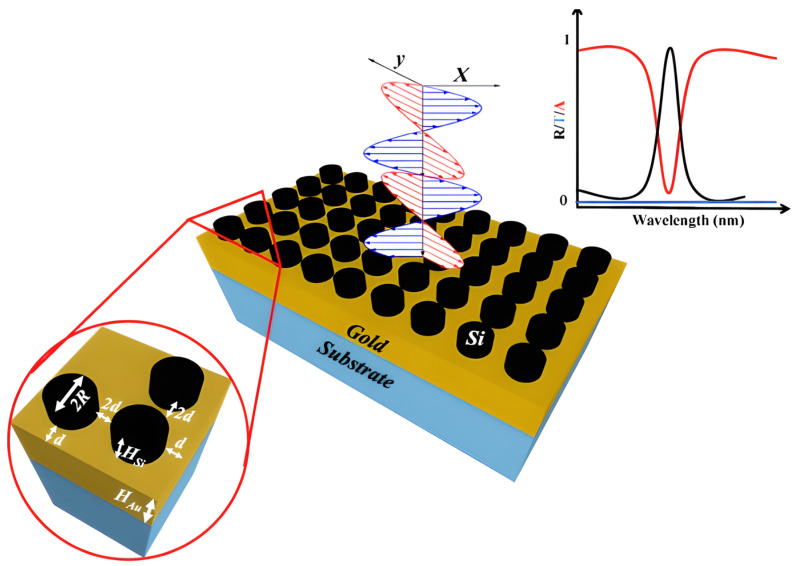
A schematic of a narrowband perfect absorber (PA) designed with a silicon nanocylinder metasurface. The inset (**bottom left**) illustrates the periodic arrangement of silicon meta-atoms (MAs) on a gold layer, while the inset (**top right**) presents the corresponding transmission, reflection, and absorption spectra [16].

Metasurfaces, composed of periodic nanoantennas, are a powerful way to detect gas because they generate strong, localized resonant fields. Functionalization is often used to render these devices more selective. For example, a CO_2_ sensor was created from silicon nanocylinders on a gold substrate and covered with a very thin layer of the PHMB polymer, as illustrated in Figure 18 [16]. When the polymer detects gas, its refractive index changes, which changes the resonance of the metasurface, thus allowing gas detection with a sensitivity of 17.3 pm/ppm [16].

A recent study [137] showed that an all-dielectric silicon metasurface working in the NIR has an ultra-high Q-factor and a sensitivity of 2100 nm/RIU. These characteristics render it perfect for finding tiny changes in the refractive index of gas molecules. The metal-insulator-metal (MIM) perfect absorber is another well-known design.

#### 4.2.5. Plasmonic Hybrid Structures

**Figure 19:**
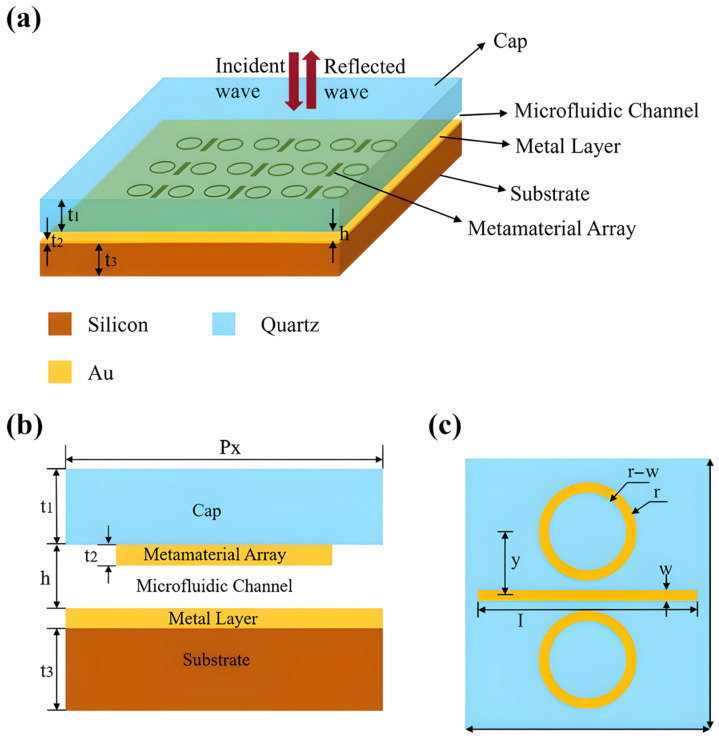
A schematic of a plasmonic hybrid sensor. (**a**) A schematic of the three-dimensional structure; (**b**) a 2D schematic of the sensor; (**c**) a structural illustration of the resonant unit [139].

Plasmonic-based resonator structures achieve good results. For example, a plasmonic perfect absorber made from a copper (Cu) grating was shown to support seven different narrowband resonances in the NIR. This multifunctional platform for on-chip gas sensing [138] enjoys a high Q-factor (10^5^) and a refractometric sensitivity of 1991.3 nm/RIU. Another study [139] suggested a dual-band NIR metasurface sensor utilizing a MIM structure for the detection of VOCs, as illustrated in Figure 19, highlighting its capability for selective, multi-gas analysis through the observation of its unique absorption peaks.

### 4.3. Interferometer-Based Photonic Gas Sensors

MZIs are a primary structure of highly sensitive phase-based gas detection. A MZI works by splitting an incoming beam of light into two paths: a sensing arm that is open to the gas environment and an isolated reference arm. When gas molecules interact with the sensing arm’s evanescent field, the arm’s effective refractive index changes, resulting in a phase shift (Δ*ϕ*) relative to the light in the reference arm. When the two beams are put back together, this phase shift changes the interference pattern, resulting in measurable changes in the output power [37]. MZI-based sensing is not only highly sensitive (with a reported LoD of 1.6 ppb for ethanol), but it is also resistant to common-mode noise from the light source, making it perfect for use in real-world applications [37]. The following subsections discuss significant advances in photonic interferometer gas sensors, grouped by the primary materials used.

#### 4.3.1. Silicon on Insulator

**Figure 20:**
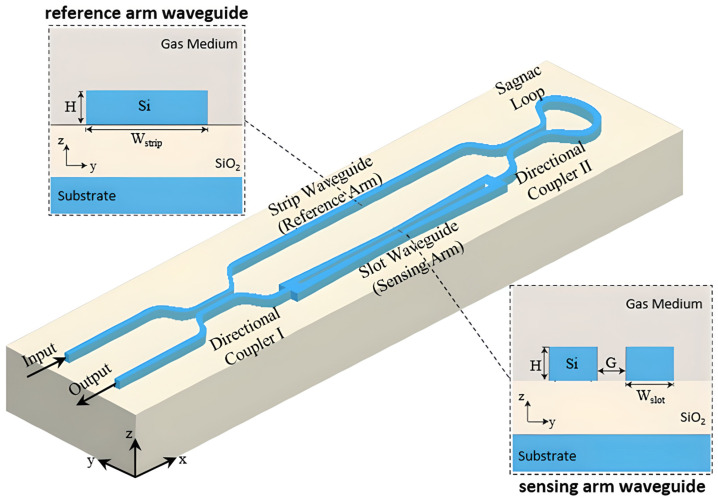
A 3D schematic of the LT-MZI gas sensor with insets showing cross-sectional views of the strip waveguide (reference arm) and slot waveguide (sensing arm) [38].

**Figure 21:**
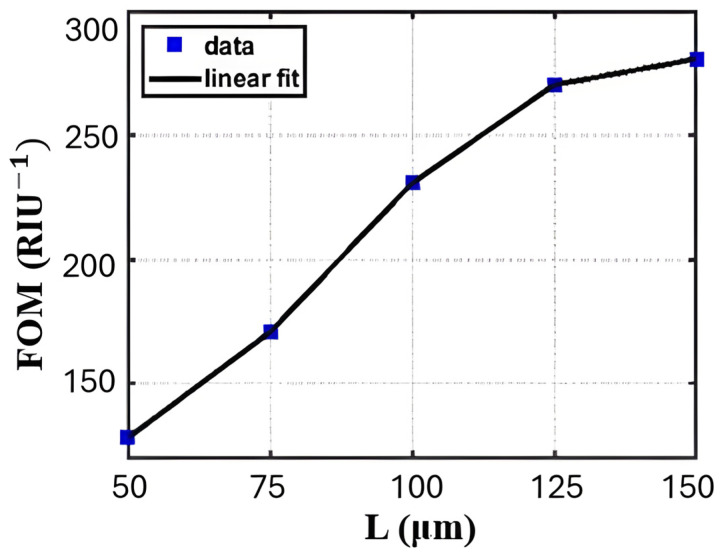
The FOM of the LT-MZI sensor as a function of interferometer arm length [38].

Structural engineering of MZIs with the mature CMOS-compatible SOI platform is key for improving gas-sensing performance in the NIR. One of the most significant challenges is that long sensing arms are needed to achieve high sensitivity, which makes the device larger. The loop-terminated MZI (LT-MZI) is a new concept that uses a Sagnac loop at the end of the arms to reflect light, as illustrated in Figure 20 [38]. The architecture effectively doubles the optical path length for a given physical size, achieving a high FOM of 280.8 RIU^−1^ in a compact 150 µm arm length, twice that of a standard MZI, as shown in Figure 21 [38]. The LT-MZI architecture uses a high-interaction slot waveguide for the sensing arm and an isolated strip waveguide for the reference arm. The design principles were successfully tested with a passive oxide-clad device that matched simulations [38]. In a recent study, an asymmetric loop-terminated MZI (a-LT-MZI) based on the idea of combining compact loops with high-interaction waveguides was introduced [140]. It further enhances sensitivity by using a Sagnac loop for compactness and adding a subwavelength grating (SWG) waveguide to the sensing arm. This design improves the interaction between light and matter to give a simulated refractometric sensitivity of 510 nm/RIU [140]. A different approach to improve interaction without loops is to change the sensing arm itself. Another study [141] suggested a multi-slot subwavelength grating MZI on the SOI platform. The device combines the strong evanescent field of multiple slots with the dispersion engineering of a SWG structure to create a highly sensitive device for sensing refractive index. Another sensor which utilizes SOI-based MZIs has also been used to detect volatile organic compounds such as ethanol and acetone. They are good for real-time monitoring in healthcare, industrial safety, and indoor air quality assessment [142] because of the fast response time and high sensitivity.

#### 4.3.2. Graphene and 2D Materials

**Figure 22:**
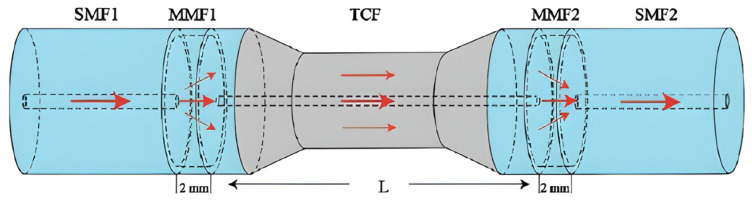
A schematic of the MZI structure incorporating a multimode-fiber–thin-core-fiber–multimode-fiber (MMF–TCF–MMF) [41].

**Figure 23:**
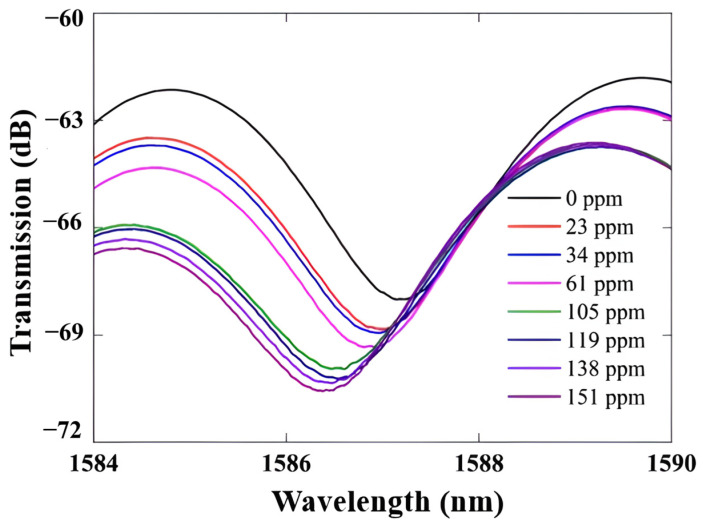
The variation in the transmission spectrum of the sensor with ammonia gas concentrations from 0 to 151 ppm [41].

2D materials are perfect for fabricating MZI-based sensors because they have a very high surface-to-volume ratio [41,143]. Target gas molecules adsorbed onto the 2D material act as charge donors or acceptors, modulating its complex refractive index and inducing a measurable phase shift in the MZI [41,143]. An all-fiber MZI is a common type of MZI. It is formed of a tapered thin-core fiber (TCF) spliced between two multimode fibres (MMFs) and covered with a layer of graphene oxide (GO), as shown in Figure 22. It could detect ammonia (NH_3_) with a linear response and have high sensitivity, as illustrated in Figure 23 [41]. A strong MZI design created from two 3 dB long-period fiber gratings (LPFGs) covered in graphene was also used to sense ammonia (NH_3_) [144]. Researchers are looking into hybrid materials that would further enhance sensitivity. A recently developed fibre-based MZI sensor [145] utilized a nanocomposite of gold (Au) nanoparticles and GO as the sensing layer. It was able to detect ammonia at the LoD of 151 ppm.

Table 3 shows some of the most significant performance metrics from the research that was covered in this work. This table delivers a clear picture of the state-of-the-art in photonic gas sensing. This comparison shows the characteristics of different approaches by comparing different material platforms (like Silicon-on-Insulator, Silicon Nitride, and polymers) and device architectures (like waveguides, resonators, and interferometers). The table shows the trade-offs and capabilities of each technology by listing the specific target gases and the level of sensitivity that can be achieved. For example, resonator-based systems have high sensitivity, while interferometric designs are more durable.

These performance metrics show how sensitive and flexible current photonic platforms are, but there are still some issues with stability, selectivity, and packaging that need to be resolved before they can be used widely in the real world. These issues will be discussed in the following section.

## 5. Challenges and Future Perspectives

Despite the advantages stated earlier, there are still several issues that need to be addressed before PIC sensors can be used to their full potential.

### 5.1. Challenges and Limitations

It is difficult to use photonic gas sensors in real life because they are not selective enough [26]. One of the issues is that other gases could alter the results and trigger false alarms. To be selective, sensors often need to distinguish the target gases from other gases that could be present. Working in the MIR range provides a built-in spectroscopic selectivity by targeting unique gas ‘fingerprints’; however, practical on-chip realization remains difficult due to high material absorption and complex light source integration. Many high-performance NIR platforms use refractive index sensing, which could respond to other gas analytes and lack chemical specificity. The widespread use of functional coatings, such as the PHMB polymer for CO_2_ sensing, is driven by the need for chemical specificity. To overcome the built-in cross-sensitivity, target recognition becomes possible by two main surface mechanisms: physical adsorption (physisorption), which depends on weak, highly reversible van der Waals and dipole forces but does not have strict specificity, and chemical adsorption (chemisorption), which uses strong, targeted molecular interactions (like covalent or hydrogen bonds) to bind only to certain analytes [146]. To differentiate individual gases in complex, multi-component gas mixtures, current integrated platforms overcome single-sensor constraints by utilizing either multiplexed sensor arrays, termed “photonic noses”, that employ pattern recognition algorithms to separate cross-interfering signals, or on-chip spectrometers that directly identify unique optical absorption fingerprints [147].

It can be hard to keep these functional layers stable, and they can break down over time [148]. Changes in temperature and humidity may render selectivity much harder because they can change how the sensor reacts to different gases. Environmental humidity may lead to polymer coatings swelling or competing for receptor sites, which can cause significant baseline drift. In addition, the useful life of these sensors is mostly limited by the irreversible degradation of these functionalized layers, rather than by the strong photonic chip itself [18].

Beyond surface coatings, another major concern is how hard it is to fabricate the gas sensor. Using CMOS-compatible platforms such as SOI and silicon nitride is a major step toward producing low-cost sensors in large quantities. To develop integrated photonic circuits that can reliably and accurately detect gases, though, advanced manufacturing methods are required. Another problem is how to ensure that the quality and performance are the same for all fabricated devices. It is still a significant engineering challenge to integrate different parts, such as light sources, waveguides, detectors, and gas-sensitive materials, into a single device without sacrificing performance. The packaging of these hybrid systems, which need to have strong electrical, optical, and thermal connections, is often an issue that raises the final cost and makes it harder to produce large photonic devices [149,150].

Once these devices are fabricated and packaged, stability is essential for photonic gas sensors to properly perform over an extended lifetime. One of the issues is that sensor responses change over time. It could happen because the sensor parts wear out, the detector surface becomes contaminated, or the sensors are used in different kinds of weather. Drift makes sensors less accurate and less sensitive [151], requiring recalibration from time to time. The sensitivity of resonant structures to changes in the temperature of the environment is a major cause of this instability. The absolute LoD, on the other hand, is directly affected by system noise caused by the laser source fluctuations, photodetector dark currents, and thermal drift within the optical cavity [18]. Photonic gas sensors can be stabilized with more robust components, coatings, and encapsulation. These measures will help sensors function the same way for a long time and reduce the effects of environmental factors [152].

The LoD is a significant challenge for photonic gas sensors, as it is difficult to detect small amounts of target gases. It is essential to achieve low LoDs, especially for safety or environmental monitoring. However, certain factors may limit the LoD of photonic gas sensors. These include noise inherent to the sensor system and interference from background gases. Additionally, VIS–NIR gas sensing depends on weak overtone absorption bands; simply adding functional groups to the surface is not enough to achieve ultra-low LoD. To strengthen light-matter interactions further, more advanced structural mechanisms need to be added. First, high-Q structures, such as micro-ring resonators, improve cavity performance by trapping photons and increasing the effective interaction length without increasing the device footprint. The Vernier effect is one of the mechanisms that can make this sensitivity even stronger. By cascading two resonators with free spectral ranges (FSR) that do not match, small changes in the gas-induced index can produce significant changes in the overall spectral envelope’s wavelength [153,154]. In addition, slow-light propagation in structures like photonic crystal waveguides cuts the guided mode’s group velocity. This spatial compression of optical energy maximizes the times over which photons and gas interact [154]. Using extended effective interaction length, Vernier-amplified shifts, or reduced group velocity compensates for the VIS–NIR regime’s weak absorption cross-sections.

Ultimately, to further improve the LoD, these structural enhancements must be combined with selecting appropriate sensing materials, using advanced signal processing to enhance the signal-to-noise ratio and reducing the sensor sensitivity to gases other than the target one [18,151].

### 5.2. Future Perspectives

Even with these issues, integrated photonic gas monitors have a bright future. Nanomaterials and metamaterials are two examples of new materials that can improve the performance of sensors. These materials can be rendered more sensitive and selective by having them react with specific gas molecules. Combining photonic sensors with other technologies, such as microelectromechanical systems (MEMS) and microfluidics, may yield small, robust, and real-time monitoring platforms capable of performing many different tasks [21,155].

Advanced computational techniques like adjoint sensitivity analysis (ASA) can produce non-intuitive designs of gas sensors [156]. Through two field simulations, ASA can efficiently calculate the gradient of an FOM with respect to all design parameters, no matter how many there are, using only two simulations [157]. Arfin et al. [158] showed how powerful this method is by designing a quad-spectral metasurface router for red, green, blue, and near-infrared (RGB-NIR) sensing. The adjoint-optimized router had an average optical efficiency of about 39% over a wide range (400–850 nm) and a very small footprint of 2 μm × 2 μm, which was much better than regular color filters. Using adjoint-based inverse design methods may accelerate the development of small and powerful photonic gas sensors [159].

A major trend shaping the future of this field is the integration of artificial intelligence (AI) and machine learning (ML). AI is no longer just a way to look at data after it has been collected. It is now a key part of the sensor system itself. By training algorithms on the complex [156], high-dimensional data produced by sensor arrays, also known as “photonic noses” [160], ML can perform advanced pattern recognition to detect and measure multiple gases simultaneously. The cross-sensitivity limits of each sensor element are thereby bypassed. AI can also correct sensor drift and temperature changes in real time, making the system more stable over time. The goal is to achieve “in-sensor computing,” which involves integrating AI algorithms directly into the photonic chip. The outcome will be self-sufficient and smart sensor nodes [25,161].

Integrated photonic gas sensors could change the way we do things across many fields, such as healthcare, environmental monitoring, and industrial process control. For instance, they could help address environmental and public health problems by providing real-time, on-site air quality monitoring with accuracy never achieved before. These sensors could be used in factories to continuously monitor dangerous gases, providing a safer workplace and greater productivity. In healthcare, they could detect biomarkers in a person’s breath, providing a new way for early disease detection without laboratory tests. Another interesting area of research is developing flexible, wearable sensors. These sensors are produced from polymers or 2D materials. They could be used to create personalized environmental monitors that humans can wear. The principles of quantum mechanics may, in the future, enhance sensor performance beyond conventional limits. Using non-classical states of light, such as squeezed light, could reduce photon shot noise and improve the signal-to-noise ratio in experiments. It would render it possible to find phenomena at the very edges of physics [18,162,163].

## 6. Conclusions

This review studied more than 140 publications focused on integrated photonic gas sensors operating in the VIS–NIR spectrum. To the best of our knowledge, this is the first review to fully investigate the integration of advanced materials science and device engineering within this practical spectral range. We discussed the basic sensing mechanisms, different material platforms (like Silicon-on-Insulator, Silicon Nitride, Chalcogenides, and 2D hybrids), as well as significant architectures like microring resonators and interferometers. Changing from bulk optics to chip-scale PICs has made it possible to detect parts-per-billion levels of gas absorption cross-sections, but problems with long-term stability and scalability remain. To solve the widespread problem of cross-sensitivity while facilitating development, multiplexed arrays of cascaded micro-ring resonators and dielectric metasurfaces on mature, CMOS-compatible Silicon-on-Insulator and Silicon Nitride platforms are the most effective technologies. Using these highly scalable structures as “photonic noses” covered in different functional polymers, it is possible to accurately separate complex gas mixtures. On-chip arrayed waveguide grating spectrometers are another solution. They offer a fully silicon-integrated method to resolve unique overtone absorption fingerprints without using coatings. Combining these optical detectors with AI and machine learning will greatly improve their performance in the future. The result will lead to smart, self-driving sensors that make real-time environmental monitoring available for everyone.

## Figures and Tables

**Table 1:** An overview of material platforms for integrated photonic gas sensing.

Material Platform	Refractive Index (n)	Transparency Window	Fabrication Constraints	Advantages	Disadvantages
Silicon-on-Insulator (SOI)	3.45	NIR (esp. 1.3–1.6 µm)	Mature CMOS-compatible fabrication	High index contrast for optical confinement; low cost; highly scalable	Indirect bandgap renders it highly inefficient for active light emission
Silicon Nitride (Si_3_N_4_)	Lower than Silicon	VIS to shortwave MIR (≈6.7 µm)	CMOS-compatible	Ultra-low propagation losses; excellent thermal stability; broad transparency	Lower refractive index contrast compared to pure silicon
InP and III–V Semiconductors	Material dependent	VIS to MIR	Complex integration with silicon	Direct bandgap enables highly efficient active light emission	Extremely difficult to mount/integrate seamlessly onto passive silicon chips
Hybrid platforms (Graphene and 2D Materials)	Variable (Modulated via charge transfer)	VIS to NIR	Requires physical transfer onto bulk waveguides	Exceptional surface-to-volume ratio; extreme sensitivity to surface perturbations	Zero intrinsic bandgap (graphene); rapid environmental degradation without passivation
Plasmonics (Au, Ag, Al)	Variable (Metal/Dielectric interface)	VIS to NIR	Requires precise nanoscale patterning	Generates strong, highly localized electromagnetic fields; enhances light-matter interaction	Certain metals lack long-term chemical stability
Lithium Niobate (LN)	Modulated via applied electric field	Ultraviolet to MIR	Scalable, high-quality thin-film fabrication	Strong Pockels electro-optic effect for ultra-fast modulation; exceptional thermal/mechanical stability	High fabrication complexity for thin-film LNOI architectures
Polymers (PMMA, SU-8)	≈1.48 (PMMA) to 1.57 (SU-8)	VIS to NIR	Low-temperature spin-coating, hot embossing, and nanoimprinting	Extremely low cost; highly flexible; easy to integrate with functional chemical coatings	Inherently lower optical confinement, often requiring hybrid integration to match silicon
Chalcogenide Glasses (As_2_S_3_)	2.4	VIS to >10 µm (Deep MIR)	Can be deposited at low temperatures without requiring lattice matching to CMOS chips	Large third-order nonlinearity; excellent broadband MIR transparency	Requires highly optimized etching processes to minimize propagation losses

**Table 2:** A structural comparison of photonic gas sensor architectures.

Device Architecture	Fundamental Physics	Advantages	Disadvantages	Footprint and Complexity	Limiting Factors
Waveguide-Based (e.g., Slot, SWG)	Evanescent Field: Gas interacts with the mode’s tail, altering absorption or refractive index	Broadband operation Simple fabrication Direct absorption measurement	Requires long interaction lengths due to weak VIS–NIR absorption	Footprint: Large Complexity: Low	Propagation and scattering losses
Resonator/Filter (e.g., Microrings)	Resonance Shift: Gas adsorption changes the optical cavity, shifting the resonant wavelength	Ultra-high sensitivity (LoD) High Q-factor enhancement	Highly sensitive to temperature drift Requires precise tunable lasers	Footprint: Ultra-compact Complexity: Moderate	Sidewall roughness
Interferometer (e.g., MZI)	Phase Shift: Refractive index changes in the sensing arm induce a measurable phase difference	High phase sensitivity Immune to laser intensity noise Highly linear response	Requires long sensing arms Difficult to fully isolate the reference arm	Footprint: Large Complexity: High	Mechanical vibrations and physical stress

**Table 3:** A comparative analysis of key performance metrics for various photonic gas sensors.

Ref. No.	Material Platform	Device Architecture	Sensing Gas	Sensitivity	Standardized LoD
[111]	SOI	Slotted Bragg grating waveguide	CO_2_	14.4 pm/ppm	Not specified (Simulated down to 215 ppm)
[113]	${Si}_{3}N_{4}$	Slot and strip waveguide	Different gases	1320 nm/RIU	Not specified (Reports FOM of 641 RIU^−1^)
[46]	Chalcogenide Glass	Waveguide	CH_4_	N/A	23 ppm
[116]	Lithium Niobate (LN)	Rib waveguide	CO_2_	High	870 ppm
[96]	PMMA with a ZIF-8 (MOF) coating	Ridge Waveguide	CO_2_	N/A	50 × 10^3^ ppm
[120]	Graphene	Waveguide	NO_2_	High	0.15 ppm
[134]	SOI	Microring	CO_2_	0.9 pm/ppm	700 ppm
[130]	${Si}_{3}N_{4}$	Racetrack Ring Resonator	Different gases	116.3 nm/RIU to 143.3 nm/RIU	Not specified (Max Q-factor: 7701)
[132]	All-polymer (SU-8)	Whispering Gallery Mode (WGM) Microdisk Resonator	Pentanoic Acid (and other VOCs)	23 pm/ppm	0.6 ppm
[16]	Metasurface (Silicon nanocylinders on a gold layer)	Metasurface-based perfect absorber microdisks	CO_2_	17.3 pm/ppm	215 ppm
[38]	SOI	Loop-terminated Mach–Zehnder Interferometer (LT-MZI)	Different gases	1070 nm/RIU	Not specified (Reports FOM of 280.8 RIU^−1^)
[41]	Graphene Oxide (GO)	MZI with a hybrid MMF-TCF-MMF structure	NH_3_	4.97 pm/ppm	151 ppm

## Data Availability

No new data were created or analyzed in this study. Data sharing is not applicable to this article.
